# Association of Feeding Methods and *Streptococcus mutans* Count with Early Childhood Caries: A Cross-sectional Study

**DOI:** 10.5005/jp-journals-10005-1420

**Published:** 2017-06-01

**Authors:** Deepa Bullappa, Manjunath P Puranik, KR Sowmya, T Nagarathnamma

**Affiliations:** 1 Senior Lecturer, Department of Public Health Dentistry, Bangalore Institute of Dental Sciences, Bengaluru, Karnataka, India; 2 Professor and Head, Department of Public Health Dentistry, Government Dental College and Research Institute, Bengaluru, Karnataka, India; 3 Assistant Professor, Department of Public Health Dentistry, Government Dental College and Research Institute, Bengaluru, Karnataka, India; 4 Professor and Head, Department of Microbiology, Bangalore Medical College & Research Institute, Bengaluru, Karnataka, India

**Keywords:** Early childhood caries, Feeding methods, *Streptococcus mutans.*

## Abstract

**Introduction:**

Early childhood caries (ECC) is a virulent form of dental caries that can destroy the primary dentition of toddlers and preschool children. The aim was to determine the relationship of feeding methods and oral *Streptococcus mutans* count in 3- to 5-year-old children with ECC.

**Materials and methods:**

A cross-sectional study was conducted in children aged 3 to 5 years. Participating mothers were interviewed regarding child’s demographic profile, educational level and socioeconomic status of parents, past medical and dental history of the mother and child, child’s feeding habits, and dietary habits and oral hygiene practices of mother and child. Clinical examination for dental caries was done using the World Health Organization criteria (1997). Salivary samples of mother-child pair were collected to determine the pH, flow rate, and S. *mutans* count. Statistical tests, such as Student’s t-test, analysis of variance, and Pearson’s correlation were applied.

**Results:**

Out of 150 mother-child pair, statistically significant difference in the caries experience was found between mothers and children with high and low S. *mutans* count. Moderate but statistically significant negative correlation was found between mean decayed, missing, and filled teeth of mothers and mean decayed, extracted and filled teeth (deft) of children with high S. *mutans* count. Regarding deft, there was no statistically significant difference between children who were exclusively breast fed (7.85 ± 2.94), exclusively bottle-fed (8.67 ± 3.98), and both breast and bottle-fed (7.77 ± 2.91).

**Conclusion:**

The mean caries experience of mothers and children was 2.66 ± 2.01 and 7.82 ± 2.94 respectively, with decayed component being maximum. Moderate and significant correlation (r = 0.5) was found between S. *mutans* of mothers and children in saliva. Significant negative correlation was found between mothers and children with high S. *mutans* count (r = -0.0284; p = 0.046).

**How to cite this article:**

Bullappa D, Puranik MP, Sowmya KR, Nagarathnamma T. Association of Feeding Methods and *Streptococcus mutans* Count with Early Childhood Caries: A Cross-sectional Study. Int J Clin Pediatr Dent 2017;10(2):119-125.

## INTRODUCTION

Early childhood caries (ECC) has been a major public health problem over many years and still continues today, affecting in many ways normal growth and development as well as social adaptation of young children. Despite the recent advances in understanding the interaction of factors that may be responsible for the development of the disease, dental caries in preschool children remains a problem for the dental clinician.^1^

The concepts of ECC and severe ECC (S-ECC) have been used for nearly 13 years to describe caries status present in children younger than 6 years. Early childhood caries is a chronic, transmissible, infectious disease with a complex and multifactorial etiology. Factors attributed to the etiology of ECC include excessive bottle-feeding with sugar-containing liquids; breastfeeding on demand and/ or falling asleep while feeding; and nursing beyond the recommended age for weaning. Other factors associated with ECC include genetic predisposition; parental education; and nutritional, environmental, socioeconomic, and parental-style factors.^2^

Although the adverse feeding patterns associated with ECC are well described, the maternal psychosocial and cultural factors underlying these behaviors that place a child at risk for ECC are unclear.^3^ There is considerable evidence that the bacterial species mutans streptococci (MS) are the principal microorganisms associated with ECC, and that MS are transmitted from mother to child. There is also evidence that maternal salivary levels of MS are associated with MS infection rates in their children aged 4 and 5 years.^1^

Hence, a cross-sectional study was undertaken with the objective to assess the feeding methods and *Streptococcus mutans* with ECC among 3- to 5-year-old schoolchildren of Bengaluru city.

## MATERIALS AND METHODS

A cross-sectional study was conducted in children aged 3 to 5 years and their mothers from January 2013 to June 2013 who can be accessed at preschools/play homes. Ethical clearance was obtained from the Institutional Ethical Committee of Government Dental College and Research Institute, Bengaluru. Necessary permission was obtained from the respective school authorities. Written informed consent was obtained from mothers of the children after explaining the study in detail.

### Sample Size

Based on the previous literature, with prevalence of dental caries in mothers as 55%, statistical power 80%, 95% confidence interval, 15% of marginal error, the sample size was computed as 150. Hence, a sample size of 150 mother-child pair was considered.

### Sampling of Schools

From the list of schools and play homes, schools were selected based on convenience sampling to facilitate transport of saliva samples within limited time to the Department of Microbiology laboratory, Bangalore Medical College and Research Institute (BMCRI), Bengaluru. Permission was sought from the school authorities for the study. Their cooperation was sought before finalizing the inclusion of the schools in the study and all children satisfying inclusion and exclusion criteria were considered.

### Inclusion Criteria

- Children within the age group of 3 to 5 years with ECC.
- Children and mothers who are willing to participate and give their consent.

### Exclusion Criteria

- Children and mothers who are medically compromised.
- Children who have lost their mothers at the time of birth.
- Children having difficulty in opening the mouth.

Mothers completed a structured questionnaire survey that included family sociodemographics [age, sex, and socioeconomic status (SES)], maternal pregnancy and past medical history (mode of delivery, gestational age, birth weight), as well as child’s feeding practice (breastfeeding, bottle-feeding, night-time feeding), dietary habits, and oral health practices of mothers and children. Clinical examination was carried out by a single trained and calibrated examiner under natural light using mouth mirror and community periodontal index probe, and recording was done by a trained assistant. Socioeconomic status was recorded according to Kuppuswamy’s SES scale updated for January 2013.^4,5^

### Obtaining and Culturing the Salivary Samples of the Participating Mother-Child Pair

Saliva collection was scheduled after the clinical examination. Preexisting saliva was allowed to swallow in order to clear the mouth of any residual unstimulated saliva. Mother and child were given a standard piece of paraffin wax to chew for 1 minute. Once the participants could chew comfortably on the wax, they were asked to expectorate all saliva in sterile disposable cups. The saliva collected was measured using 1 mL of tuberculin syringe.

The saliva samples were assessed for pH and flow rate; pH was checked using pH strips (coding of 2-10). Flow rate of ≤0.2 mL/minute was considered low and 0.3 to 0.7 mL/minute was considered as normal.^6^

The saliva samples were transported to the Department of Microbiology, BMCRI, Bengaluru, using thio-glycolate media (transportation media) within a span of 2 hours for culturing into the prepared agar plates on the same day. Codes were given to the syringes with a permanent marker as to differentiate the samples. Before the saliva samples were cultured, the media plates were incubated at temperature of 37°C for 30 minutes, to remove the moisture from the plates. Serial dilutions (1:1,000) of saliva samples were done with distilled water; 0.1 mL of saliva was transferred to 9.9 mL of distilled water in sterilized test tube. The test tube was given the same code as on the syringe from which the saliva was transferred. The mixture was stirred and 0.1 mL of this was taken. A standard inoculation loop (4 mm inner diameter) was used to transfer exactly 0.1 mL and it was streaked on mitis salivarius bacitracin agar, selective for MS prepared according to Gold et al.^7^ The area on plate was divided into two halves using a glass marker and was coded with the same numbers as on the respective test tube from which the saliva was transferred. The cultured plates were immediately transferred to the carbon dioxide jar and incubated for 48 hours at 37°C. The colonies were counted using magnifying lens and recorded as N × 10^4^ colony-forming units (CFU) per mL of saliva. These colonies were classified into low (<50 × 10^4^ CFU) and high (>50 × 10^4^ CFU).

### Statistical Analysis

The data collected were entered into Excel spreadsheet and analyzed using the Statistical Package for Social Sciences version 16.0. Descriptive statistics with frequency, percentage, mean, and standard deviation were taken. Statistical significance was considered at p < 0.05 (confidence interval of 95%). Statistical tests, such as Student’s t-test, analysis of variance, and Pearson’s correlation were applied.

**Table 1:** **Table 1:** Distribution of the study groups (children) according to feeding practices

		*N(%)*		*Total*	
*Feeding method*					
Breastfeeding exclusively		29 (19.3)		150 (100)	
Bottle-feeding exclusively		6 (4)			
Breastfeeding and bottle-feeding		115 (76.7)			
*Duration of breastfeeding*					
Birth to 6 months		26 (18.05)		144 (100)	
Birth to 12 months		62 (43.05)			
Birth to 24 months		48 (33.34)			
Birth to more than 24 months		8 (5.56)			
*Feeding practice at bedtime*					
Breastfeeding		46 (30.7)		150 (100)	
Bottle containing liquids (other than		22 (14.7)			
water)					
Both breast- and bottle-feeding		41 (27.3)			
Neither breast nor bottle-feeding		41 (27.3)			
*Feeding practice during night*					
Breastfeeding/bottle-containing liquid		104 (69.3)		150 (100)	
other than water					
Comforting the child/a pacifier/a bottle		3 (2)			
containing water					
Comforting the child		43 (28.7)			
*Frequency of feeding at night*					
One or two times		83(79.8)		104 (100)	
Three to seven times		19 (18.3)			
Throughout night		2 (1.9)			

**Table 2:** **Table 2:** Distribution of the study groups (mothers and children) according to the oral hygiene practices and dietary practices

		*Mothers* *N(%)*		*Children* *N(%)*	
*Oral hygiene aid used*					
Toothbrush		150(100)		142 (94.7)	
Finger		0 (0)		8 (5.3)	
Other aids		0 (0)		0 (0.0)	
*Method of cleaning*					
Vertical		71 (47.3)		17 (11.3)	
Horizontal		62 (41.3)		106 (70.7)	
Circular		17 (11.3)		27 (18)	
*Material used for cleaning*					
Toothpaste		149 (99.3)		149 (99.3)	
Tooth powder		1 (0.7)		1 (0.7)	
Any other		0 (0)		0 (0)	
*Frequency of cleaning*					
Once		110 (73.3)		120 (80)	
Twice		37 (24.7)		28 (18.7)	
More than twice		3 (2)		2 (1.3)	
*Time of brushing*					
Before meals in the morning		104 (69.3)		110 (73.3)	
After meals at night		3 (2)		8 (5.3)	
Both times		43 (28.7)		32 (21.3)	
*Frequency of sugar intake*					
Seldom or never		0		4 (2.7)	
Once a day		20 (13.3)		33 (22)	
Twice a day		100 (66.7)		76 (50.7)	
Thrice a day		23 (15.3)		30 (20)	
Four times or more		7 (4.7)		7 (4.6)	

## RESULTS

Out of 150 children, 74 (49.3%) were males and 76 (50.7%) were females, with the mean age of 4.33 ± 0.79 years, and the mean age of mothers was 29.51 ± 2.91 years. Majority [87 (58%)] of the study groups belonged to upper middle class.

Out of 150 children, 112 (74.7%) were born vaginally and 38 (25.3%) by cesarean section. More than 50% of the mothers [97 (64.67%)] cleaned children’s gum pads after feeding, wherein 54 (55.67%) cleaned with damp clean cloth (Table 1).

With regard to cleaning of their child’s teeth [133 (88.7%)] mothers cleaned always; 4 (2.7%) cleaned sometimes, and 80 (53.3%) introduced cleaning at the age of 12 months. The present study showed that more than 75% were born at normal term with normal birth weight (123 [82%]). Tables 2 and 3 show the distribution of study groups according to oral hygiene, dietary practices, and salivary characteristics respectively.

**Table 3:** **Table 3:** Cross-tabulation of study groups according to salivary characteristics

		*pH: Child*					
*pH: Mother*		*Acidic n (%)*		*Neutral n (%)*		*Basic n (%)*	
Acidic		12 (66.67)		6 (33.33)		0	
Neutral		36 (28.8)		89 (71.2)		0	
Basic		1 (14.3)		2 (28.6)		4 (57.1)	
*Flow rate: Mother*				*Flow rate: Child*		
				*Low n (%)*		*Normal n (%)*	
Low (≤0.2 mL/minute)				8 (80)		2 (20)	
Normal (0.3-0.7 mL/minute)				17 (12.1)		123 (87.9)	
*S. mutans: Mother*				S. mutans: Child*			
				*Low n (%)*		*High n (%)*	
Low (<50 × 10^4^ CFU)				64 (63.36)		37 (36.64)	
High (>50 × 10^4^ CFU)				5 (10.2)		44 (89.8)	

r = 0.5, *p = 0.00

Statistically significant correlation was found between decayed (DT/dt), filled (FT/ft), and decayed, missing, and filled teeth (DMFT)/decayed, extracted and filled teeth (deft) of mothers and children, whereas weak and nonsignificant correlation was found for MT/et (Table 4).

Statistically significant difference was found between mean deft of children having low and high *S. mutans* count, whereas moderate but statistically significant negative correlation was found between mean DMFT of mothers and mean deft of children with high *S. mutans* count (Table 5).

Regarding deft, there was no statistically significant difference between children who were exclusively breast fed (7.85 ± 2.94), exclusively bottle-fed (8.67 ± 3.98), and both breast and bottle-fed (7.77 ± 2.91).

Statistically significant difference was noted for caries experience among low and high *S. mutans* count children who were both breast and bottle-fed and breast fed exclusively. Weak and significant correlation was found between the deft of children having high *S. mutans* count who were breast fed and children who were both breast-and bottle-fed (Table 6).

**Table 4:** **Table 4:** Mean caries experience (DMFT/deft) among study groups (mother-child)

		*Mothers*		*Children*		*Correlation* * coefficient*	
DT/dt		1.89 ± 1.71		7.18 ± 2.86		0.32*	
MT/et		0.37 ± 0.69		0.37 ± 0.99		0.10	
FT/ft		0.41 ± 0.69		0.27 ± 0.83		0.28*	
DMFT/deft		2.66 ± 2.01		7.82 ± 2.94		0.44*	

*Highly significant

**Table 5:** **Table 5:** Mean caries experience among study groups (mother-child) according to *S. mutans count*

		*Mothers* *(mean DMFT)*		*Children* *(mean DMFT)*	
Low *S. mutans* count		1.74 ± 1.30		5.81 ± 1.62	
High *S. mutans* count		4.55 ± 1.89^a^		9.53 ± 2.72^b,c^	

^a^significant difference between mothers with high and low S. *mutans* count (p = 0.00); **S**ignificant difference between children with high and low S. *mutans* count (p = 0.00); Significant negative correlation between mothers and children with high *S. mutans.* S. *mutans* count (r = -0.0284), (p = 0.048)

**Table 6:** **Table 6:** Mean caries experience among study group (children) according to S. *mutans* count and feeding practices

		*Breast fed* *(mean deft)*		*Bottlefed* *(mean deft)*		*Both* *(mean deft)*	
Low S. *mutans* count		6.20 ± 1.78		5		5.72 ± 1.59	
High S. *mutans* count		9.64 ± 2.25^a^		9.4 ± 3.975		9.52 ± 2.62^b,c^	

^a^Significant difference between low and high S. *mutans* count breast fed children (p = 0.001); **S**ignificant difference between low and high S. *mutans* count both breast- and bottle-fed children (p = 0.00); Significant weak correlation between breast and both breast- and bottle-fed children with high *S. mutans* count (r = 0.215; p = 0.046)

## DISCUSSION

In the present study, the age of the mothers ranged from 22 to 38 years, with the mean age being 29.51 ± 2.91 years, which is in line with some studies,^8-12^ whereas the mean age of children in the present study is 4.33 ± 0.79 years, which is comparable to some studies.^13-15^

The present study consisted of nearly equal proportion of males and females (49.3:50.7). This is in line with three studies (49.7:50.3),^16^ (51:49),^17^ (278:252),^18^ whereas in some studies, there was a predominance of males over females^8,17-30^ and in other studies, females over males.^31-33^

Income was updated using All India’s Average Consumer Price Index for Industrial Workers (CPI-IW) for January 2013. To modify income, the conversion factor (221) between the CPI-IW for 1998 and January 2013 was determined.^4^ With a total of 150 in the study group, maximum, i.e., 87 (58%), belonged to upper middle class (II). A study conducted among Anganwadi children in Wardha, India, showed that 60.3% participants belonged to social class V; 34.5% participants belonged to social class IV.^31^ These results differ from the present study as there was a difference in the participants’ social background.

Neonatal factors may also increase the risk for early acquisition of *S. mutans* via vertical transmission. Infants delivered by cesarean section acquire *S. mutans* earlier than vaginally delivered infants.^34^ In the present study, about three-fourths [112 (74.7%)] were born by vaginal delivery, whereas one-fourth [38 (25.3%)] was born by cesarean section. In an Indian study,^35^ 87.1% were delivered normally. In a study^28^ where mode of delivery was considered as a risk factor for ECC, 184 out of 350 were vaginally born.

Regarding the mode of delivery, it is in line with a study where 65.4% were born ≥37 weeks of gestational period.^28^ Regarding birth weight, one study^19^ reported significant proportion of low birth weight (36.5%) and in another study, 3.7%^28^ reported ≤2,500 g.

There is some debate as to whether infant formula or bovine milk in bottles and breast milk given frequently to young children contribute to the development of ECC.^36^ In this study, more than three-fourths of the children [115 (76.7%)] were both bottle- and breast fed. In studies reported, breastfeeding ranged from 5 to 100%.^11,14,18,19,28,31,33,37-39^ Bottle-feeding was widely prevalent in most of the studies ranging from 51 to 97.9%,^13,17,21,22,38,40-43^ whereas maximum mixed feeding (both breast and bottle-feeding) was found in some studies, ranging from 19 to 42.6%.^20,27,32,35,44^

About 50% of the mothers cleaned their child’s gum pads after feeding with damp clean cloth. This is in line with a study conducted in Guntur where 71%^32^ of the mothers cleaned after feeding, whereas in contrast, another study reported that 11.4%^33^ cleaned after feeding. Regarding the material used to clean gum pads, the result of this study is in contrast with the study where 52% used finger to clean gum pads after feeding^32^ and similar to the studies (11.4^33^ and 19%^32^) that used cotton/gauze.

Toothbrushing behavior is learned from models as part of the socialization process. Because parents play a key role in the family in transferring health-related habits to the children, their toothbrushing has been associated with oral cleaning frequency of their children.^45^ These results with regard to aidedtooth brushing are in line with the studies where proportion of aided brushing ranged from 72 to 97%,^32,42,46^ while other studies have reported lesser proportion (39^18^-57.5%^16^) of aided brushing by mothers as fathers/caregivers were also involved in aided brushing.

Some studies have reported that children of mothers who had a dietary preference for sweets had more ECC compared with children whose mothers had a nonsweet preference. These studies suggest that a mother’s personal preferences play an important role in shaping her child’s dietary preference for sugar and thus can influence child’s risk for ECC.^47^ The present study emphasized on these lines where almost equal proportion of mothers and children consumed sugar twice daily.

In this study, mother-child pairs shared similar pH status to a greater extent in acidic, neutral, and basic groups, more so among neutral group. Acidic-basic combination and neutral-basic combination among mother-child pairs was not observed, while 1 (14.3%) had basic-acidic combination.

Comparing salivary flow rate, mother-child pair shared similar salivary flow rate to a greater extent in low and normal groups, more so among normal group. The ratio of finding similar flow rate to dissimilar flow rate was 4:1 and 7:1 for low and normal salivary flow rate respectively, among mother-child pair.

Out of 101 (100%) mothers having low *S. mutans* count, 64 (63.36%) of their children had low *S. mutans* count and 37 (36.64%) of their children had high count. Similarly, among mothers [49 (100%)] with high count, 5 (10.2%) of their children had low count and 44 (89.8%) of their children had high *S. mutans* count. In a study,^10^ in the group of mothers with high salivary *S. mutans* counts (> 150 CFU), 52% of children were colonized with the bacteria. Conversely, the proportion of mothers with low *S. mutans* counts (1-15 CFU) was > 15%.

The study showed moderate but statistically significant (p = 0.048) negative correlation between mean DMFT of mothers (4.55 ± 1.89) and mean deft of children - (9.53 ± 2.72) with high *S. mutans* count. In one study,^48^ higher salivary counts of *S. mutans* (≥10^5^ CFU/mL) have been correlated with high dmft values (11.5 ± 3.2) and the difference was statistically significant. In another study^49^ having greater than 50 CFUs it was also significantly associated (p = 0.03) with the presence of ECC, with a higher proportion of those with greater than 50 CFUs having ECC. A study showed a positive correlation between ECC and the levels of *S. mutans* counts (p = 0.034).^9^

Method of feeding is shown to be associated with rampant caries with higher prevalence occurring in children who were never breast fed.^20^

The present study showed no statistically significant difference among the mean scores of all three groups (F = 0.269, p = 0.765). This is in line with the studies with regard to breastfeeding and caries where there was no statistically significant difference found.^40^

### Limitations of the Study

The study sample comprised children attending private preschools/play-homes representing a certain class of society. Further studies may include children attending Anganwadis to have a wider representation. This study considered children with ECC for determining its association with feeding methods and *S. mutans.* Further studies may compare children with ECC/S-ECC and without ECC regarding feeding methods and *S. mutans.* The microbial culture methods employed detects growth of MS group. Hence, further studies may consider specific *S. mutans* media if feasible.

### Recommendations

The alarming finding in this study concerning the practice of bottle-feeding in children at night points to the need to provide mothers with instructions and guidance in the feeding methods and subsequent harmful snacking patterns.

Mothers should be educated at prenatal stage about maintenance of child’s oral health by cleaning the gum pads every time after feeding with damp clean cloth/ cotton/gauze.

As young children lack the ability to clean their own teeth effectively, parents should clean their children’s teeth with fluoridated toothpaste at least until they reach school age.

This study showed correlation of mothers and child’s *S. mutans* level in saliva. Hence, mothers with high levels of cariogenic bacteria must be identified and their bacterial levels should be reduced prior to infant tooth eruption in an effort to delay and/or reduce the cariogenic bacteria levels in their infants.

Mothers should be instructed to use the lift-the-lip technique to spot the white-spot lesions as first signs of dental caries.

Parents should realize that they are role models for their children. Parents’ own oral health behaviors and their active role in performing oral cleaning for their children should be emphasized in dental and general health settings.

## CONCLUSION

The mean caries experience of mothers and children was 2.66 ± 2.01 and 7.82 ± 2.94 respectively, with decayed component being maximum. Moderate and significant correlation (r = 0.5) was found between *S. mutans* of mothers and children in saliva. Significant negative correlation was found between mothers and children with high *S. mutans* count (r = -0.0284; p = 0.046).
